# *EVI1* promotes tumor growth via transcriptional repression of *MS4A3*

**DOI:** 10.1186/s13045-015-0124-6

**Published:** 2015-03-21

**Authors:** Gerwin Heller, Anna Rommer, Katarina Steinleitner, Julia Etzler, Hubert Hackl, Petra Heffeter, Erwin Tomasich, Martin Filipits, Birgit Steinmetz, Thais Topakian, Simone Klingenbrunner, Barbara Ziegler, Andreas Spittler, Sabine Zöchbauer-Müller, Walter Berger, Rotraud Wieser

**Affiliations:** Department of Medicine I, Medical University of Vienna, Währinger Gürtel 18-20, 1090 Vienna, Austria; Comprehensive Cancer Center of the Medical University of Vienna, Vienna, Austria; Biocenter, Division of Bioinformatics, Medical University of Innsbruck, Innrain 80, 6020 Innsbruck, Austria; Department of Medicine I, Institute of Cancer Research, and Research Platform “Translational Cancer Therapy Research”, Borschkegasse 8A, 1090 Vienna, Austria; Core Facility Flow Cytometry & Surgical Research Laboratories, Medical University of Vienna, Währinger Gürtel 18-20, 1090 Vienna, Austria

**Keywords:** EVI1, MS4A3, Transcriptional repression, Myeloid leukemia, Tumor growth

## Abstract

**Background:**

The transcription factor Ecotropic Virus Integration site 1 (EVI1) regulates cellular proliferation, differentiation, and apoptosis, and its overexpression contributes to an aggressive course of disease in myeloid leukemias and other malignancies. Notwithstanding, knowledge about the target genes mediating its biological and pathological functions remains limited. We therefore aimed to identify and characterize novel EVI1 target genes in human myeloid cells.

**Methods:**

U937T_EVI1, a human myeloid cell line expressing *EVI1* in a tetracycline regulable manner, was subjected to gene expression profiling. qRT-PCR was used to confirm the regulation of membrane-spanning-4-domains subfamily-A member-3 (*MS4A3*) by EVI1. Reporter constructs containing various parts of the *MS4A3* upstream region were employed in luciferase assays, and binding of EVI1 to the *MS4A3* promoter was investigated by chromatin immunoprecipitation. U937 derivative cell lines experimentally expressing *EVI1* and/or *MS4A3* were generated by retroviral transduction, and tested for their tumorigenicity by subcutaneous injection into severe combined immunodeficient mice.

**Results:**

Gene expression microarray analysis identified 27 unique genes that were up-regulated, and 29 unique genes that were down-regulated, in response to EVI1 induction in the human myeloid cell line U937T. The most strongly repressed gene was *MS4A3*, and its down-regulation by EVI1 was confirmed by qRT-PCR in additional, independent experimental model systems. *MS4A3* mRNA levels were also negatively correlated with those of *EVI1* in several published AML data sets. Reporter gene assays and chromatin immunoprecipitation showed that EVI1 regulated *MS4A3* via direct binding to a promoter proximal region. Experimental re-expression of *MS4A3* in an *EVI1* overexpressing cell line counteracted the tumor promoting effect of *EVI1* in a murine xenograft model by increasing the rate of apoptosis.

**Conclusions:**

Our data reveal *MS4A3* as a novel direct target of EVI1 in human myeloid cells, and show that its repression plays a role in *EVI1* mediated tumor aggressiveness.

**Electronic supplementary material:**

The online version of this article (doi:10.1186/s13045-015-0124-6) contains supplementary material, which is available to authorized users.

## Background

Overexpression of the *E*cotropic *V*irus *I*ntegration site 1 (*EVI1*) gene, which has been observed in subsets of patients with acute myeloid leukemia (AML) [1-4], myelodysplastic syndromes (MDS) [5-7], chronic myeloid leukemia (CML) [8-10], and certain solid tumors [11-14], is often associated with poor therapy response and shortened survival [1-4,7,9,11,12,15,16]. In mouse bone marrow transduction/transplantation models, experimental expression of *Evi1* led to development of an MDS-like disease [17], or to AML-like disease when co-expressed with other oncogenes [18,19]. It also enhanced the growth of xenograft tumors in severe combined immunodeficient (SCID) mice [20]. *In vitro*, *EVI1* stimulated cellular proliferation and inhibited differentiation and apoptosis in some experimental models [14,17,20-29], but evoked opposite responses in others [17,29-37], indicating that the consequences of *EVI1* overexpression may be influenced by cell lineage, maturation stage, cooperating molecular events, and/or environmental stimuli. EVI1 is believed to exert its varied biological functions predominantly by regulating gene transcription, and recently large-scale approaches have been applied to identify its target genes in ovarian cancer and murine myeloid cell lines [38,39]. A limited number of genes were shown to be regulated by EVI1 in a direct manner and to contribute to some of its biological effects, e.g., *Gata2* [24], *Pbx1* [40], *Pten* [41], *Gpr56* [42], *miR-1-2* [43], *miR-9* [44], *miR-124* [45,46], and *miR-449A* [47]. In light of the multitude of cellular responses to EVI1, however, its target genes and mechanisms of action are still far from completely understood.

The membrane-spanning 4-domains subfamily A member 3 (*MS4A3*) gene was expressed in specific subsets of hematopoietic cells, including myeloid precursors, basophilic granulocytes, and CD34-positive hematopoietic stem and progenitor cells induced to differentiate *in vitro* by exposure to granulocyte colony stimulating factor (G-CSF) [48-50]. MS4A3 was present in a complex with cyclin-dependent kinase 2 (CDK2) and kinase-associated phosphatase (KAP), which inactivates CDK2 by dephosphorylation of Thr160 [50]. MS4A3 stimulated the enzymatic activity of KAP, and caused cell cycle arrest when expressed in human myeloid U937 cells in a regulable manner [50,51].

In the present study, we found that *MS4A3* was repressed by EVI1 in several experimental model systems. This repression was mediated by direct binding of EVI1 to a proximal region in the *MS4A3* promoter, and was necessary for the tumor promoting effects of *EVI1* in a murine xenograft model.

## Results

### *MS4A3* is repressed strongly and rapidly in response to induction of EVI1

**Figure 1:**
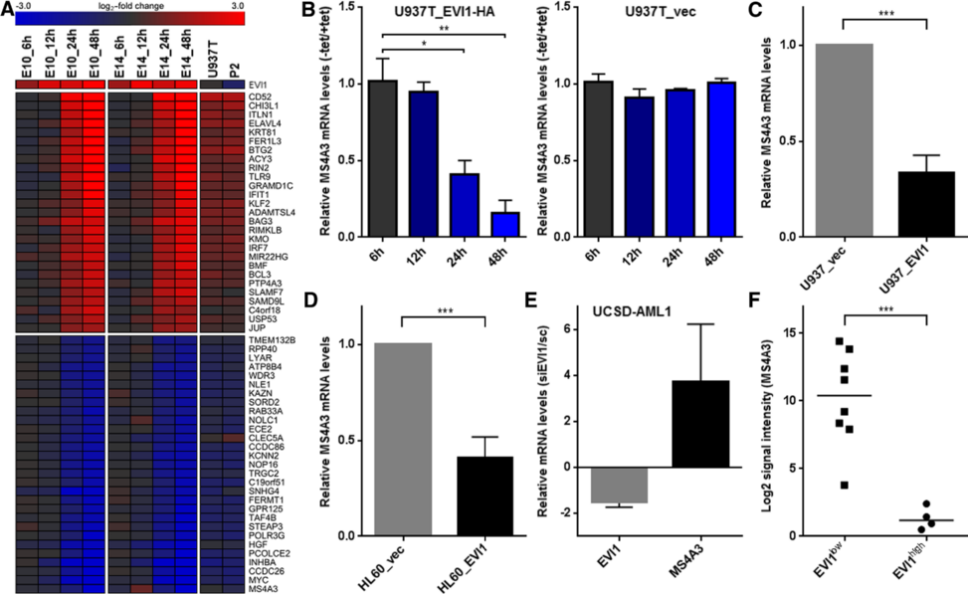
***MS4A3*** **is strongly repressed by EVI1 in human myeloid cells. A)** Heatmap summarizing expression changes of 56 genes affected by induction of EVI1 in U937T_EVI1-HA cells (clones E10 and E14) as determined by microarray analyses at different time points after transfer to tetracycline (tet) free media. Parental U937T cells and U937T_vec (clone P2) cells incubated with or without tet for 48 h were used as controls. Log_2_ transformed expression changes relative to cultures maintained in the presence of tet (red, upregulated; blue, downregulated) are shown in descending order. **B)** qRT-PCR confirmed repression of *MS4A3* in U937T_EVI1-HA, but not U937T_vec cells after tet withdrawal. **C, D)** qRT-PCR showing EVI1-mediated down-regulation of *MS4A3* in U937 **(C)** or HL-60 **(D)** cells constitutively expressing ectopic *EVI1*. **E)** qRT-PCR showing induction of *MS4A3* after siRNA mediated down-regulation of EVI1 in UCSD-AML1 cells. Data in B-E represent means + SEMs from at least three independent biological replicate experiments. **F**) *MS4A3* mRNA levels in a panel of 12 human myeloid cell lines (8 with low and 4 with high *EVI1* expression) represented in GEO data set GSE35159 [54]. *p < 0.05; **p < 0.01; ***p < 0.001 (Student’s t-test, two-tailed). The induction of *MS4A3* after knock-down of EVI1 in UCSD-AML1 cells was not significant, but an at least 1.8-fold up-regulation was observed in four out of four independent biological replicate experiments.

We have previously established U937T_EVI1-HA clones E10 and E14, which express an HA epitope-tagged version of the human *EVI1* cDNA in a tetracycline (tet) repressible manner in the background of the human myeloid cell line U937 [34]. In E10 and E14 cells, expression of the EVI1 protein is strongly induced as early as 12 h after tet withdrawal, is sustained for at least 120 h, and its peak levels are comparable to those in HNT-34 cells [34], which express endogenous EVI1 due to a rearrangement of its gene locus at 3q26 [52]. In order to identify genes whose mRNA levels were altered rapidly in response to induction of EVI1, E10 and E14 cells were cultured in the absence or presence of tet for 6, 12, 24, and 48 h, RNA was extracted, converted to cRNA, and hybridized to Human Genome U133 Plus 2.0 arrays (Affymetrix). As controls, parental U937T cells and empty vector transfected U937T_vec (clone P2) cells that had been incubated with or without tet for 48 h were processed in the same manner. Tet withdrawal affected gene expression patterns not only in EVI1-expressing, but also in control cells [53]. Consequently, only those genes were considered to be regulated by EVI1 whose mRNA levels changed at least 2-fold 48 h after tet withdrawal in both E10 and E14 cells, and whose induction or repression at this time point exceeded any background effects observed in either U937T or P2 cells as described in the Methods section. According to these criteria, 56 unique genes were found to be responsive to EVI1: 27 genes were up-, and 29 genes were down-regulated subsequent to the induction of this transcription factor (Figure 1A). Gene ontology (GO) analysis revealed significant enrichment of the terms “cytokine biosynthetic process” and “regulation of apoptosis” among the EVI1-regulated genes (Additional file 1: Table S1). The gene most strongly induced by EVI1 in this system was *CD52*, which has previously been shown to be up-regulated by EVI1 and proposed as an immunotherapeutic target for *EVI1*-positive leukemia [54]. On the other hand, the most strongly (~16-fold) repressed gene was *MS4A3*, which has been reported as a negative regulator of the mitotic cycle of hematopoietic cells [50], and was also strongly repressed in response to inducible ectopic expression of Evi1 in primary murine hematopoietic cells [29]. Even though a region ~4 kb from the transcriptional start site of the murine *Ms4a3* gene was found to be bound by EVI1 in a ChiP-seq screen [39], the precise mechanistic basis and biological consequences of the repression of *MS4A3* by EVI1 have so far not been investigated.

To corroborate the microarray results, RNA extracted from U937T_EVI1-HA and U937T_vec cells maintained in the presence or absence of tet for 6 to 48 h was subjected to reverse transcriptase qRT-PCR. These experiments confirmed that *MS4A3* was repressed strongly, rapidly, and specifically upon induction of EVI1 (Figure 1B). Similarly, *MS4A3* was significantly down-regulated in U937_EVI1 [20] and HL-60_Evi1 [55] cells, which experimentally express EVI1 in a constitutive manner, as compared to the respective empty vector transduced control cells (Figure 1C, D). Conversely, siRNA mediated knockdown of EVI1 in UCSD-AML1 cells, which express high endogenous levels of this gene [56], led to up-regulation of *MS4A3* (Figure 1E). In additional support of the regulatory relationship between EVI1 and *MS4A3*, analysis of gene expression omnibus (GEO) microarray data sets GSE35159 [54], GSE6891 [57], GSE14471 [58], and GSE35784 [59] revealed that human myeloid cell lines and primary AML patient samples with high *EVI1* mRNA levels exhibited low expression of *MS4A3* and vice versa (Figure 1F, Table 1).

**Table 1:** **Negative association between** ***EVI1*** **and** ***MS4A3*** **mRNA levels in primary samples from AML patients**

**Data set**	**Cutoff**	**# EVI1** ^**high**^	**# EVI1** ^**low**^	**% EVI1** ^**high**^	**M**	**SD**	**Z**	***P*** **-Value**
GSE6891 C1	5.97	18	229	7.3	-1.36	0.40	3.37	7.4E-04
GSE6891 C2	6.16	12	202	5.6	-1.16	0.54	2.16	3.1E-02
GSE14471	8.61	9	102	8.1	-1.86	0.73	2.56	1.0E-02
GSE35784	5.66	21	109	16.2	-1.01	0.36	2.84	4.5E-03

Bootstrap analysis was performed on GEO microarray data sets GSE14471 [58], GSE35784 [59], and GSE6891 [57], the latter of which consists of two independent patient cohorts (C1, C2). For each of the four patient cohorts, cutoff values defining high (EVI1^high^) versus low (EVI1^low^) EVI1 expression were determined as described in Materials and Methods. The respective groups of EVI1^high^ patients were compared to 10.000 randomly sampled, equally sized groups of EVI1^low^ patients. Cutoff, log_2_ intensity of *EVI1* expression defining EVI1^high^ versus EVI1^low^ patients; M, mean difference of log_2_ transformed *MS4A3* expression between EVI1^high^ and randomly permuted EVI1^low^ patients; SD, standard deviation of M; Z, Z-score of the sampling distribution (Z = -M/SD).

### EVI1 regulates *MS4A3* through a promoter proximal region

**Figure 2:**
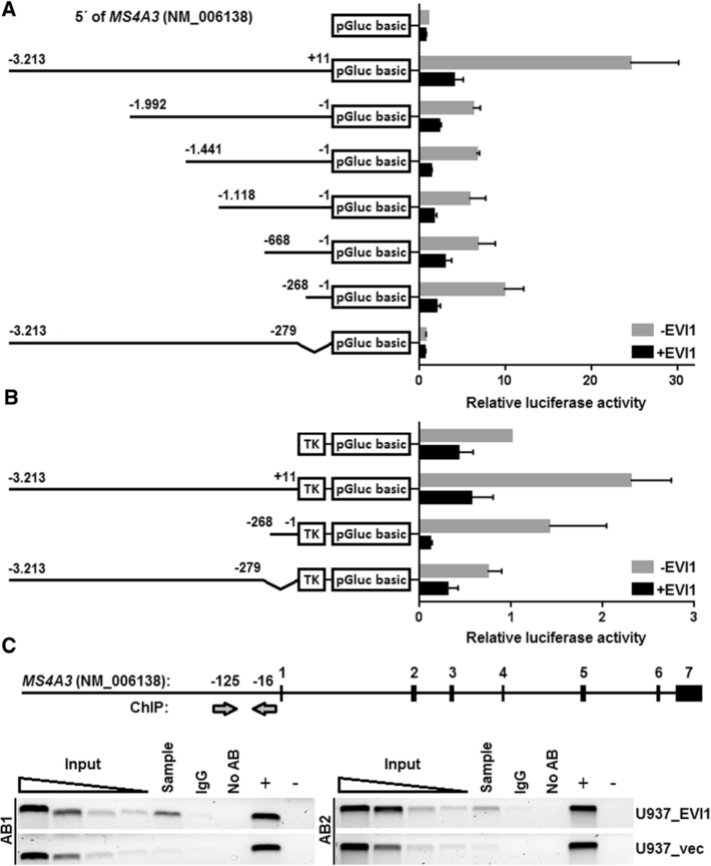
**EVI1 regulates** ***MS4A3*** **by directly binding to a proximal element in its promoter. A)** Luciferase assays with *MS4A3* promoter deletion constructs. The *MS4A3* 5′ region, starting from -3213 relative to the transcription start site, and several 5′ deletion variants thereof were cloned into the promoterless Gaussia luciferase reporter vector, pGluc basic. Reporter plasmids and either an EVI1 expression vector (+EVI1; black bars) or empty vector as a control (-EVI1; grey bars) were transfected into U937 cells, and luciferase activity was measured from cell supernatants two days later. pGluc basic without any *MS4A3* 5′ sequences was used as negative control. **B)** Similar experiments were performed using some of the above described reporter plasmids with the HSV tk basal promoter inserted between the *MS4A3* 5′ regions and the luciferase gene of pGluc basic. Data in A) and B) represent means + SEMs from three independent biological replicate experiments. **C)** ChIP assays were performed on U937_EVI1 and U937_vec cells using two different EVI1 antibodies (AB1, sc-8707X, Santa Cruz; AB2, C50E12, Cell Signaling). Primers used for ChIP PCR amplified a region in the proximal *MS4A3* promoter as indicated by the arrows in the upper panel. IgG, negative control using nonspecific IgG; no AB, negative control without antibody; +, input DNA (positive control); -, H_2_O (negative) PCR control.

To test whether EVI1 would affect the *MS4A3* promoter in a direct manner, and to identify potential EVI1-responsive regions, reporter gene assays were performed. An approximately 3.2 kb fragment representing the upstream region of the human *MS4A3* gene was cloned into the promoterless Gaussia luciferase reporter vector, pGluc basic, to yield pMS4A3(-3213/+11)/pGluc. 5′ deletion variants of this vector were prepared in an analogous manner. The reporter plasmids were transfected into U937 cells, along with an *EVI1* expression vector or empty vector as a control. As shown in Figure 2A, all *MS4A3* reporter constructs, including pMS4A3(-268/-1)/pGluc, were repressed by EVI1, suggesting that EVI1 acted directly on the *MS4A3* promoter, and that the 268 proximal base pairs were sufficient to mediate this effect. Deletion of this region from the full length reporter vector yielded pMS4A3(-3213/-279)/pGluc. The absence of repression by EVI1 indicated that the proximal region of the *MS4A3* promoter was not only sufficient, but also necessary for the response to EVI1 (Figure 2A). To ensure that the loss of regulation by EVI1 was not simply a consequence of the removal of basal promoter elements, and therefore to a general expression defect in pMS4A3(-3213/-279)/pGluc, equivalent vectors were generated, but with the HSV tk promoter inserted between the *MS4A3* upstream regions and the pGluc sequences. As shown in Figure 2B, pMS4A3(-3213/+11)/tk/pGluc, containing the 3.2 kb *MS4A3* upstream sequence in front of the tk promoter, was repressed by EVI1. An even stronger effect was observed with pMS4A3(-268/-1)/tk/pGluc, which comprised only the EVI1-responsive proximal promoter region, whereas removal of this region in pMS4A3(-3213/-279)/tk/pGluc reduced the repression by EVI1 to basal levels.

To confirm that EVI1 bound to the relevant region of the *MS4A3* promoter in intact cells, chromatin immunoprecipitation (ChIP) was performed (Figure 2C). Two different EVI1 antibodies, but not an isotype control antibody, recovered substantial amounts of DNA that could be amplified with oligonucleotide primers specific for the proximal region of the *MS4A3* promoter from U937_EVI1 cells. Confirming the specificity of the assay, no such enrichment was observed when U937_vec cells were used.

In summary, these data suggest that EVI1 regulates expression of *MS4A3* by directly binding to a DNA element located upstream of and in close vicinity to its transcriptional start site, and that this region is both necessary and sufficient to mediate repression by EVI1.

### Experimental re-expression of *MS4A3* in *EVI1*-positive cells counteracts the acceleration of tumor growth effected by *EVI1*

Having identified *MS4A3* as a gene that was regulated by EVI1 in a direct manner and in several independent experimental systems, we next asked whether repression of *MS4A3* contributed to cellular phenotypes elicited by EVI1. To this end, the human *MS4A3* cDNA was cloned into pMIA-II, a retroviral vector containing the fluorescent marker gene Ametrine. U937_vec and U937_EVI1 cells were infected with empty pMIA-II as a control or with pMIA-II_MS4A3, yielding the cell lines U937_vec_vec, U937_vec_MS4A3, U937_EVI1_vec, and U937_EVI1_MS4A3. Cells were sorted for Ametrine positivity, and the expression of MS4A3 according to the expected pattern was verified by immunofluorescence analysis (Additional file 2: Figure S1). Since EVI1 had previously been shown to inhibit myelomonocytic differentiation in this experimental model system [20], the possibility that re-expression of *MS4A3* may alleviate this effect was explored. U937_vec_vec, U937_vec_MS4A3, U937_EVI1_vec, and U937_EVI1_MS4A3 cells were treated with 25-OH Vitamin D3 or solvent (EtOH) for 5 days, stained with CD11b or isotype control antibody, and subjected to flow cytometry. The results of these experiments corroborated the notion that EVI1 inhibited myelomonocytic differentiation, yet MS4A3 had no effect on this process either in the absence or in the presence of EVI1 (Additional file 3: Figure S2).

**Figure 3:**
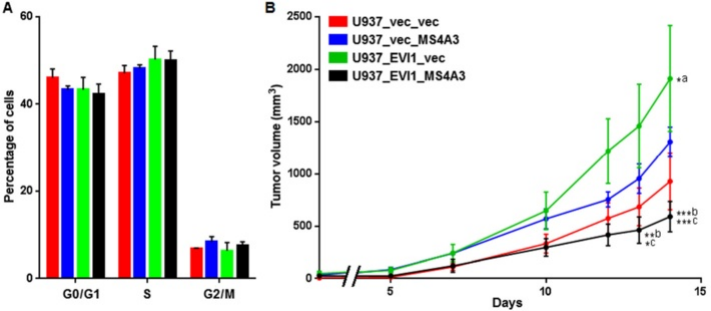
**Ectopic expression of MS4A3 counteracts the tumor promoting effect of EVI1 in a murine xenograft model. A)** Cell cycle analysis of U937_vec_vec (red bars), U937_vec_MS4A3 (blue bars), U937_EVI1_vec (green bars), and U937_EVI1_MS4A3 (black bars) cells after propidium iodide staining of nuclei isolated from cells growing exponentially in suspension culture. Data represent means + SEMs of three independent biological replicate experiments. **B)** U937_vec_vec (red line), U937_vec_MS4A3 (blue line), U937_EVI1_vec (green line), and U937_EVI1_MS4A3 (black line) cells were subcutaneously injected into SCID mice (4 animals per cell line) and tumor volume was measured at the indicated time points. *p <0.05; **p <0.01; ***p <0.001; two-way ANOVA and Bonferroni post-correction. a, U937_vec_vec vs. U937_EVI1_vec; b, U937_vec_MS4A3 vs. U937_EVI1_MS4A3; c, U937_EVI1_vec vs U937_EVI1_MS4A3.

**Figure 4:**
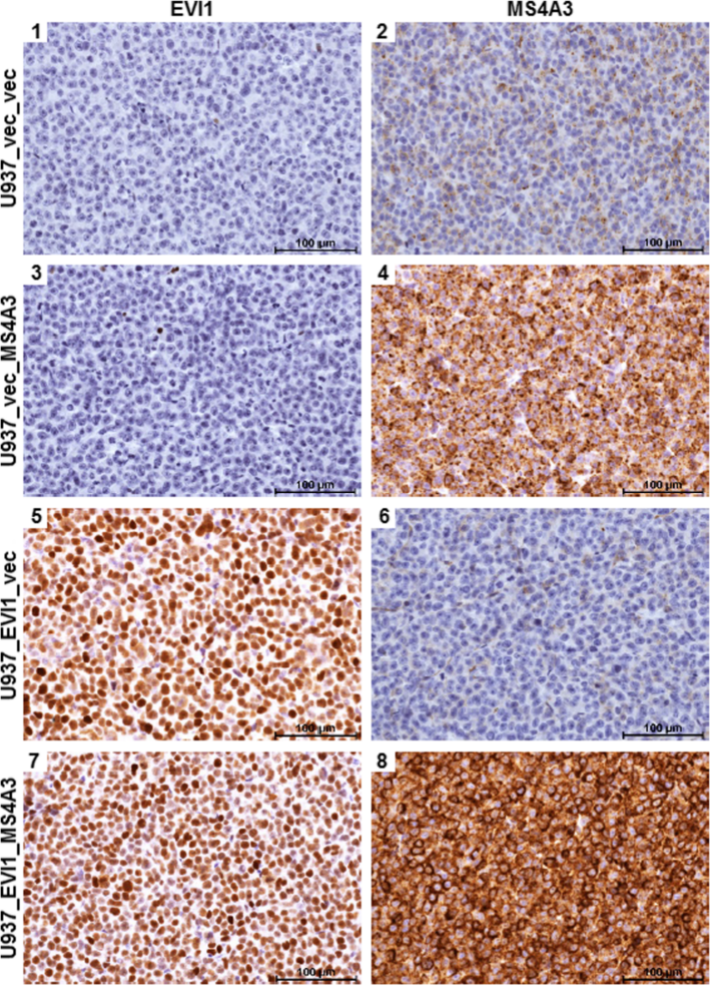
**Persistent expression of ectopic EVI1 and MS4A3 in xenograft tumors, and confirmation of down-regulation of endogenous MS4A3 by EVI1 at the protein level.** Immunohistochemical analyses of EVI1 (left panel) and MS4A3 (right panel) in xenograft tumors derived from U937_vec_vec, U937_vec_MS4A3, U937_EVI1_vec, and U937_EVI1_MS4A3 cells. Scale bar, 100 μm.

**Figure 5:**
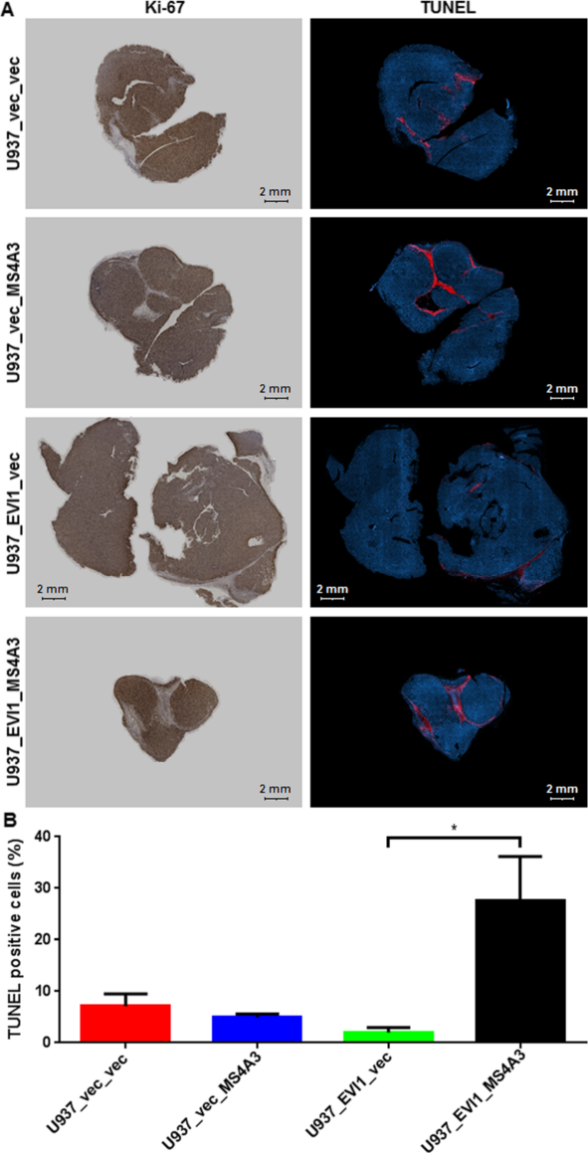
**MS4A3 enhances apoptosis in EVI1-positive xenograft tumors. A)** Whole sections of tumors derived from U937_vec_vec, U937_vec_MS4A3, U937_EVI1_vec, and U937_EVI1_MS4A3 cells were subjected to immunohistochemical staining for Ki-67 (left panel), or to staining for double strand breaks using the TUNEL method (right panel). Representative images are shown. Scale bar, 2 mm. **B)** Bar plot showing mean percentages + SEMs of TUNEL positive cells in 3 tumors of each of the 4 xenograft groups. *p < 0.05 (Student’s t-test, two-tailed).

Next, we asked whether EVI1 and/or MS4A3 would affect cellular proliferation *in vitro* or *in vivo*. Even though constitutive experimental expression of neither of these genes altered the cell cycle distribution of U937 cells *in vitro* in a significant manner (Figure 3A), EVI1 strongly and significantly enhanced the growth of tumors derived from these cells after subcutaneous injection into SCID mice, and re-expression of MS4A3 abolished this effect (Figure 3B). Immunohistochemical staining of tumor sections corroborated both the down-regulation of endogenous MS4A3 by EVI1 at the protein level, and the persistent expression of exogenous EVI1 and MS4A3 in the xenograft tumors (Figure 4). To investigate whether the observed disparity in tumor growth was attributable to different rates of proliferation and/or cell death, tumor sections were stained for the proliferation marker Ki-67 and for cell death-associated double strand breaks using the terminal deoxynucleotidyl transferase dUTP nick end labeling (TUNEL) method. These experiments showed that all tumors contained large areas that were composed almost exclusively of Ki-67-positive cells, included high proportions of mitotic figures, and were interspersed only with sporadic TUNEL-positive cells (Figure 5A, Additional file 4: Figure S3A, and data not shown). On the other hand, some fractions of the tumors comprised high proportions, or consisted almost exclusively, of TUNEL-positive, Ki-67-negative cells (Figure 5A, Additional file 4: Figure S3B). The overall percentage of TUNEL positive cells was significantly higher in U937_EVI1_MS4A3 tumors than in U937_EVI1_vec tumors (Figure 5B), suggesting that re-expression of MS4A3 in EVI1-positive myeloid cells may slow tumor growth by enhancing the rate of cell death.

## Discussion

*EVI1* is an oncogene whose overexpression is associated with high aggressiveness of both hematological and solid tumors [1-4,7,9,11,12,15,16]. Even though this correlation is well established, and the molecular structure, nuclear localization, and DNA binding ability of EVI1 suggest that it acts as a transcription factor [60], the target genes and molecular mechanisms through which it contributes to the emergence and therapy resistance of malignant diseases are still understood only to a limited extent. Recently, genome-wide large-scale approaches have been applied to identify genes regulated by EVI1 in murine hematopoietic cells and a human ovarian cancer cell line [29,38,39]. In the present study, we used a complementary approach and searched for genes whose expression levels changed in response to inducible expression of EVI1 in a human myeloid cell line. Among 56 bona fide EVI1-regulated genes, the *MS4A3* gene, coding for a member of a family of four-transmembrane proteins, was repressed most strongly after induction of EVI1. *MS4A3* was also down-regulated in primary murine hematopoietic cells inducibly expressing *Evi1* [29], and its mRNA levels changed in the expected direction after manipulation of *EVI1* expression in three additional human myeloid cell line based models (Figure 1 C-E). When CD34-positive primary human hematopoietic stem and progenitor cells were differentiated into the granulocytic lineage *in vitro*, *EVI1* levels decreased [36] while *MS4A3* levels increased [50] (and KS, unpublished results). Furthermore, *EVI1* expression was negatively correlated with that of *MS4A3* in a panel of human myeloid cell lines and in primary samples from AML patients (Figure 1F, Table 1). Reporter gene assays and ChIP showed that EVI1 regulated *MS4A3* by directly binding to the proximal 268 bp of its promoter. ChIP-seq on a murine leukemic cell line also identified an EVI1 binding site near the *Ms4a3* gene [39], yet at a greater distance from its transcriptional start site, and the functional significance of this site was not further investigated. Previous studies have defined a number of different consensus EVI1 binding sites [38,39,61-67], but interestingly, none of these sites was found in the 268 bp region delineated through the luciferase assays, suggesting that EVI1 has the ability to recognize DNA motifs in addition to those identified in these earlier studies.

To date, little is known about the biological functions of *MS4A3*. Donato et al reported that inducible expression of this gene in U937 cells retarded their re-entry into the cell cycle after release from S-phase arrest [50]. Using a constitutive overexpression approach in the same cell line, we did not observe any effect of *MS4A3* on the cell cycle distribution of asynchronously proliferating cells (Figure 3A), or on re-entry into the mitotic cycle of cells synchronized in the same manner as described by Donato et al (JE, unpublished results). Possible explanations for this divergence are the use of different expression systems and/or different U937 sublines between the Donato and our own studies. However, additional investigations will be required to resolve this discrepancy.

The reciprocal expression patterns of *EVI1* [36] and *MS4A3* [50] (and KS, unpublished results) during *in vitro* differentiation of primary human CD34-positive cells into the granulocytic lineage raise the possibility that repression of *MS4A3* may contribute to the differentiation inhibiting effect of EVI1 [17,20,29]. However, ectopic expression of *MS4A3* in U937_EVI1 or U937_vec cells did not affect their differentiation in response to 25-OH Vitamin D3 (Additional file 3: Figure S2), indicating either that induction of *MS4A3* is a consequence rather than a cause of myeloid maturation, or that other model systems are required to reveal a potential differentiation promoting effect of *MS4A3*.

A gene expression signature characterizing leukemic stem and progenitor cells as opposed to the bulk leukemic population was associated with poor outcome in AML, and low expression of *MS4A3* constituted part of this signature [68]. *MS4A3* was also significantly down-regulated in a cyclophosphamide-resistant CML cell line as compared to the corresponding parental line (GEO data set GDS2729 [69]). We therefore asked whether repression of *MS4A3* could play a role in *EVI1*-mediated drug resistance of human myeloid leukemic cells [20,27,70], yet re-expression of *MS4A3* in U937_EVI1 cells did not re-sensitize them to drugs used in the treatment of AML (JE and SK, unpublished results). Nevertheless, a role for down-regulation of *MS4A3* in *EVI1*-induced disease aggressiveness was obtained in a murine xenograft model, in which tumors formed by U937_EVI1 cells grew significantly faster than U937_vec tumors, while re-expression of *MS4A3* abolished this effect. Interestingly, *MS4A3* did not slow the growth of *EVI1*-negative U937_vec tumors, suggesting either that endogenous *MS4A3* was expressed at saturating levels in this cell line, or that *MS4A3* specifically interfered with tumor growth on the background of the gene expression pattern evoked by EVI1. The first possibility would predict that U937_EVI1_MS4A3 and U937_vec_MS4A3 grew at equal rates. The observation that in fact U937_EVI1_MS4A3 cells formed significantly smaller tumors than U937_vec_MS4A3 cells discredits the former explanation in favor of the latter. The *in vivo* phenotypes of *EVI1* and *MS4A3* are also notable in light of the absence of an effect of either of these genes on cellular proliferation in suspension cultures *in vitro*. This suggests that specific aspects of the growth condition *in vivo*, e.g., interactions with the tumor microenvironment, are required for them to reveal their impact on cell and tumor growth.

## Conclusions

In summary, our data uncover *MS4A3*, a so far poorly studied gene, as a novel direct target of EVI1 in myeloid cells, and show that its repression plays a role in *EVI1*-mediated tumor aggressiveness. These results increase the still fragmentary understanding of the way of action of *EVI1*, an oncogene that is of great clinical importance because its overexpression is associated with poor therapy response in a variety of malignant diseases.

## Methods

### Cell lines, retroviral transductions, immunofluorescence analysis, and gene knockdown

Cell lines U937T_EVI1-HA, represented by clones E10 and E14, and U937T_vec, represented by clone P2, have been described previously [34]. They were cultured in RPMI 1640 (Life Technologies, Carlsbad, CA, USA) containing 10% fetal bovine serum (FBS; Life Technologies), 0.5 μg/ml puromycin (Sigma-Aldrich, St Louis, MO, USA), 500 μg/ml hygromycin (PAA, Pasching, Austria), and 1 μg/ml tetracycline (Sigma-Aldrich) in a humidified incubator at 37°C and 5% CO_2_. To induce EVI1 expression, exponentially growing cells were washed 3 times with PBS (Life Technologies) and resuspended in growth media without tetracycline. Control cultures were washed in the same manner but were resuspended in media with tetracycline.

Cell lines U937_EVI1, U937_vec [20], HL60_Evi1, and HL60_vec [55] were grown in RPMI 1640 containing 10% FBS and 1% Penicillin/Streptomycin/Glutamine (PSG; Life Technologies). The coding sequence of the human *MS4A3* gene (transcript variant 1, NM_006138.4) was amplified using cDNA from U937_vec cells, the primers listed in Additional file 5: Table S2, and Phusion High Fidelity Polymerase (New England Biolabs, Ipswich, MA, USA). PCR products were cloned into the retroviral vector pMIA-II_IRES-Ametrine using the BamHI and XhoI sites to yield pMIA-II_MS4A3-IRES-Ametrine. DNA sequencing was performed to confirm the identity and accuracy of the insert. Retroviral particles were generated and U937_EVI1 and U937_vec cells were infected using standard procedures. After 3 days, cells were sorted for Ametrine positivity on a FACS Aria (Becton Dickinson, Franklin Lakes, NJ, USA). MS4A3 expression was confirmed by immunofluorescence analysis (IF). In brief, cells were transferred onto cover slips coated with Cell-Tak™ Cell and Tissue Adhesive (Corning Incorporated, Corning, NY) and fixed with ice-cold methanol (Roth, Karlsruhe, Germany). IF was performed using rabbit polyclonal MS4A3 antibody HPA019210 (Atlas Antibodies; dilution 1:30) and the Rhodamine (TRITC)-AffiniPure F(ab′)2 Fragment Goat Anti-Rabbit IgG (H + L) secondary antibody (Jackson ImmunoResearch, West Grove, PA, USA; dilution 1:200).

UCSD-AML1 cells [56] were maintained in RPMI 1640 supplemented with 20% FBS, 1% PSG, and 10 ng/ml GM-CSF (PeproTech, Rocky Hill, NJ). 2.25 × 10^6^ cells from a logarithmically growing culture were resuspended in 400 μl of PBS and electroporated either with a mix of EVI1 siRNAs (stealth siRNAs HSS103423 and HSS103424, Invitrogen) or with scrambled control siRNA (stealth siRNA 462001, Invitrogen) at final concentrations of 100 nM. Electroporation was carried out in a Gene Pulser Xcell Electroporation System (BioRad, Hercules, CA) at 300 V and 1000 μF using an exponential protocol. Electroporated cells were incubated under standard growth conditions for 24 h prior to RNA extraction.

### Gene expression microarrays and statistical and bioinformatics analyses

For gene expression microarray analyses, U937T_EVI1-HA E10 and U937T_EVI1-HA E14 cells were washed and placed into media with or without tetracycline for 6, 12, 24, and 48 h. To control for potential effects of tetracycline removal in the absence of EVI1 induction, U937T_vec P2 and U937T cells incubated in the presence or absence of tetracycline for 48 h were also included in the experiment. Total RNA was extracted using the RNeasy kit (Qiagen, Hilden, Germany) as recommended by the manufacturer. RNA quality control, sample labelling and hybridization to Affymetrix HG-U133 plus 2.0 microarrays (Affymetrix, Santa Clara, CA, USA) were performed at the Center of Excellence for Fluorescent Bioanalytics (KFB; Regensburg, Germany). Robust Multi-array Analysis was used for background correction, quantile normalization and median polish summarization of probe levels. Only probe sets with a current gene annotation and with average log_2_-intensities ≥3 at 24 and 48 h in E10 and E14, and at 48 h in P2 and U937T cells, were included in downstream analyses. Because we had previously observed background effects of tetracycline withdrawal in control cells [53], probe sets were considered as regulated by EVI1 only if they were induced or repressed at least two-fold both at 24 and 48 h after tetracycline withdrawal and both in E10 and E14 cells, and in addition the effect of tetracycline removal at 48 h in E10 and E14 cells was at least 10^(fold-change expression/3) the effect in the control cell lines P2 and U937T. If more than one probe set for the same gene was found to be regulated in this manner, the probe set with the most pronounced regulation was included in the heatmap, which was generated using Genesis [71]. All other computational analyses and filtering procedures were performed using R and custom PERL scripts. Microarray data were deposited in the GEO database (accession number GSE60100).

GO term enrichment was analysed using the term-for-term algorithm of Ontologizer [72]. P-values were calculated using one-sided Fisher exact test, and adjusted for multiple hypothesis testing according to Benjamini and Hochberg [73]. An adjusted p-value <0.1 was considered statistically significant.

GEO datasets GSE6891 [57], GSE14471 [58], and GSE35784 [59], which contain gene expression data from primary AML samples, were probed for differences in *MS4A3* expression between samples with high or low levels of *EVI1* by bootstrap analysis. To determine cutoff values defining high versus low *EVI1* expression, the density distributions of the log_2_ transformed EVI1 mRNA levels were estimated using a Kernel Density Estimator (KDE). The *EVI1* expression values at which the density distribution exhibited a minimum were used as cutoffs for the respective data set. In datasets where several local minima existed, the minimum closest to the EVI1^low^ distribution with <5% of the maximal density defined the cutoff. The respective groups of EVI1^high^ patients were compared to randomly sampled, equally sized groups of EVI1^low^ patients. 10.000 iterations of this setup were performed, and in each step the difference between the mean log_2_ transformed *MS4A3* expression values in both groups was calculated. Finally, the mean value of the resulting distribution (log_2_-fold change, M) and the two-sided P-value using the inverse standard normal cumulative distribution function were determined.

Differences in *MS4A3* expression between human myeloid cell lines with high or low *EVI1* mRNA levels as represented in GEO data set GSE35159 [54] were probed for significance by the CyberT algorithm of Flexarray software (http://www.gqinnovationcenter.com/downloads/index.aspx?l=e).

To predict potential binding sites of EVI1 in the *MS4A3* promoter, 19 different position frequency (weight) matrices (PWMs) were newly compiled or derived from experimentally verified binding sites [38,39,61-67] or from the Matbase matrix library 8.4 (Genomatix) and JASPAR [74] databases. Potential EVI1 binding sites in the genomic region from -268 to -1 relative to the transcriptional start site of *MS4A3* were identified based on a PERL implementation of the MatInspector algorithm [75] if the similarity score for a specific PWM was equal to or above a threshold that was defined by allowing one binding site per 10 kb of human coding sequences. Genomic sequences were derived from the UCSC genome browser [76].

### qRT-PCR

Total RNA for qRT-PCR was extracted using Trizol (Life Technologies) and reverse transcribed using random hexamer primers (Life Technologies) and M-MLV reverse transcriptase (Life Technologies) according to the manufacturer’s instructions. qRT-PCR was carried out in a Step One Plus Real Time PCR system (Applied Biosystems, Life Technologies) using standardized cycling conditions as recommended by the manufacturer. Levels of *EVI1*, *MS4A3*, and the housekeeping gene *Cyclophilin D* were determined using the primers listed in Additional file 5: Table S2 and the Mesa Green qPCR MasterMix Plus (Eurogentec, Eraing, Belgium). All assays were performed in triplicate. Expression values for the gene of interest relative to the housekeeping gene and to a reference value were determined using the ΔΔC_T_ method [77]. At least three biological replicates were analysed and mean fold changes in expression and standard errors of the mean (SEM) were calculated.

### Reporter vectors and luciferase assays

All vectors used for luciferase assays were based on pGluc basic (New England Biolabs). The *MS4A3* 5′ region (-3213/+11 relative to the transcription start site of NM_006138) was amplified from human genomic DNA using Phusion® High-Fidelity DNA Polymerase (New England Biolabs) and the primers listed in Additional file 5: Table S2. The resulting PCR product was ligated to the EcoRV digested vector, yielding pMS4A3(-3213/+11)/pGluc. A series of 5′ deletion constructs (-1992/-1, -1441/-1, -1118/-1, -668/-1, -268/-1 and -3213/-279) was generated by PCR amplification using the cloned promoter fragment MS4A3(-3213/+11) as a template, followed by subcloning using EcoRI and BamHI restriction enzymes (Fermentas Inc., Hanover, MD, USA).

pMS4A3(-3213/+11)/tk/pGluc, pMS4A3(-268/-1)/tk/pGluc, and pMS4A3(-3213/-279)/tk/pGluc were generated by subcloning the HSV tk promoter into the BamHI, or the KpnI and BamHI, sites of the respective pGluc basic based constructs.

For luciferase assays, 6 × 10^5^ U937 cells/well were seeded into 12-well plates. Transient transfections of reporter constructs and either empty pcDNA3 (Life Technologies) or pcDNA3-EVI1 (containing a codon optimized version of the human EVI1 cDNA) were performed using 1 μg DNA (reporter:effector ratio = 1:3) and 4 μl of JetPEI cationic polymer transfection reagent (Polyplus, Illkirch, France) according to the manufacturer’s instructions. After 48 h, 50 μl of culture supernatant were mixed with 50 μl of Gluc assay solution from the BioLux® Gaussia Luciferase Flex Assay Kit (New England Biolabs). The bioluminescent reaction was measured immediately by detecting the emitted photons at 475 nm using a Tristar LB941 (Berthold Technologies, Bad Wildbad, Germany). The values represent means + SEMs of three independent experiments.

### Chromatin immunoprecipitation (ChIP)

ChIP assays were performed using the chromatin immunoprecipitation assay kit (Upstate Biotechnology, Lake Placid, NY, USA) as reported previously [78]. In brief, 5 × 10^6^ U937_EVI1 or U937_vec cells were fixed by treatment with 1% formaldehyde for 10 min and then lysed. Chromatin was sheared to fragments of 200 - 1000 bp using Bioruptor (Diagenode, Liege, Belgium). Immunoprecipitation was performed using rabbit monoclonal EVI1 antibody C50E12 (Cell Signaling, Danvers, MA, USA; dilution 1:80) or rabbit polyclonal EVI1 antibody sc-8707X (Santa Cruz Biotechnology, Santa Cruz, CA, USA; 1:250). Nonspecific IgG (2729, Cell Signaling, 1:200) was used as a negative control. Immunoprecipitated DNA was extracted with phenol/chloroform (Sigma-Aldrich), precipitated with ethanol, and dissolved in 30 μl Tris-EDTA buffer (Sigma-Aldrich). 2 μl of recovered DNA were subjected to PCR analysis using the primers shown in Additional file 5: Table S2 and HotStarTaq DNA Polymerase (Qiagen). Cycling conditions were: 95°C for 12 min, followed by 32 cycles at 95°C for 30 s, 60°C for 40 s, and 72°C for 30 s, and a final incubation step at 72°C for 7 min. PCR products were separated on a 2% agarose gel stained with GelRed (Biotium, Hayward, CA, USA).

### Analyses of myelomonocytic differentiation and of cell cycle distribution

To analyse myelomonocytic differentiation of U937 cells, logarithmically growing cells were seeded to a density of 2 × 10^5^ cells/ml and incubated either with 100 nM 25-OH-Vitamin D3 (Calbiochem, La Jolla, CA) or with an equivalent amount of solvent (EtOH) for 5 days. Cells were diluted once during this period to avoid saturating densities, and fresh 25-OH-Vitamin D3 was added at the same time. After blocking of nonspecific epitopes with Human TruStain (Biolegend, San Diego, CA), cells were stained with monoclonal rat APC-Cy7 conjugated CD11b antibody (clone M1/70, Biolegend) or corresponding isotype control (clone RTK4530, Biolegend) using standard procedures. Flow cytometric analysis was performed on an LSRFortessa™ SORP (BD Biosciences, Bedford, MA, USA).

For cell cycle analyses, cells were adjusted to a density of 400 cells/μl. On the next day, cells were washed with PBS (Life Technologies) and incubated for 5 min in ice cold 0.5 M citrate/0.5% Tween-20. Cell membranes were disrupted mechanically before nuclei were pelleted and resuspended in PBS containing 100 μg/ml RNase A (Sigma-Aldrich) and 50 μg/ml propidium iodide (PI; Sigma-Aldrich). Nuclear DNA content was determined on a FACSCalibur™ (BD Biosciences) or a FACS LSRFortessa™ SORP using ModFit software (Verity Software House, Topsham, ME, USA) for data analysis.

### Xenograft experiments and immunohistochemistry

Animal experiments were approved by the ethics committee of the Medical University of Vienna and the Bundesministerium für Wissenschaft und Forschung Ref. II/10b (Gentechnik und Tierversuche), application Nr. BMWF-66.009/0095-II/10b/1010, and were carried out according to the Austrian and FELASA guidelines for animal care and protection in order to minimize distress for the animals. Mice were sacrificed by cervical dislocation.

Six to eight week old male CB-17 scid/scid (SCID) mice were purchased from Harlan Laboratories (San Pietro al Natisone, Italy). The animals were kept in a pathogen-free environment and all procedures were performed in a laminar airflow cabinet. 5 × 10^6^ U937_vec_vec, U937_vec_MS4A3, U937_EVI1_vec, or U937_EVI1_MS4A3 cells, resuspended in 50 μl of serum-free RPMI 1640 medium, were injected subcutaneously into the right flanks of 4 mice per cell line. Animals were controlled every day and tumor size was assessed regularly by caliper measurement. Tumor volume was calculated using the formula: (length × width^2^)/2. At experiment termination, mice were dissected and tumor tissue was processed for immunohistochemistry (IHC). For statistical analysis of tumor growth, two-way ANOVA and Bonferroni post-correction were applied.

IHC was performed using standard procedures. Briefly, 4 μm sections from xenograft tumor blocks were deparaffinized and rehydrated, heated for 10 min in 10 mM citrate buffer (pH 6.0) in a pressure cooker for epitope retrieval, and then incubated for 60 min at room temperature with rabbit monoclonal EVI1 (clone C50E12, Cell Signaling Technology; dilution 1:200) or rabbit polyclonal MS4A3 (HPA019210, Atlas Antibodies; dilution 1:50) antibodies, or for 30 min with mouse monoclonal Ki-67 antibody (MIB-1, Dako, Glostrup, Denmark; dilution 1:100). Antibody binding was detected by means of the UltraVision LP detection system (Lab Vision, Thermo Fisher Scientific, San Jose, CA, USA) according to the manufacturer’s recommendations. Color development was performed by 3-3′-diaminobenzidine (Dako) and counterstaining by hematoxylin (Merck, Vienna, Austria). Terminal deoxynucleotidyl transferase dUTP nick end labeling (TUNEL) was carried out using the in situ cell death detection kit, TMR Red (Roche, Mannheim, Germany) according to the manufacturer’s instructions.

Images of stained tumor sections were acquired with TissueFAXS (TissueGnostics, Vienna, Austria). Percentages of TUNEL-positive cells were determined using TissueQuest software (TissueGnostics).

## Additional files

Additional file 1: Table S1. Gene Ontology (GO) categories overrepresented among the genes regulated in response to induction of EVI1 in U937T_EVI1-HA cells. Additional file 2: Figure S1. Confirmation of ectopic expression of MS4A3 in transduced U937_EVI1 and U937_vec cells. Immunofluorescence analysis of MS4A3 expression in U937_vec_vec **(A)**, U937_vec_MS4A3 **(B)**, U937_EVI1_vec **(C)**, and U937_EVI1_MS4A3 **(D)** cells. Endogenous MS4A3 was observed in U937_vec_vec and U937_EVI1_vec cells, and strong ectopic MS4A3 expression was present in U937_vec_MS4A3 and U937_EVI1_MS4A3 cells. Additional file 3: Figure S2. EVI1 inhibits 25-OH Vitamin D3 induced myelomonocytic differentiation of U937 cells, but ectopic expression of *MS4A3* has no impact on this process. U937_vec_vec, U937_vec_MS4A3, U937_EVI1_vec, and U937_EVI1_MS4A3 cells were treated with EtOH (solvent) or 25-OH Vitamin D3 for 5 days, and the extent of myelomonocytic differentiation was determined by flow cytometry after staining for CD11b. **(A)** Histograms from a representative experiment. Blue areas, isotype control antibody; red areas, APC-Cy7 conjugated CD11b antibody. **(B)** Summary of flow cytometric data from three independent biological replicate experiments. Mean percentages of positive cells + SEMs are shown. ***p <0.001; (Student’s t-test, two-tailed). Additional file 4: Figure S3. Nuclear localization of Ki-67 and of double strand break containing DNA identified through TUNEL staining. Magnifications from one of the tumors shown in Figure 4B. **(A)** Immunohistochemical staining for Ki-67; **(B)** TUNEL staining. Scale bar, 50 μm. Additional file 5: Table S2. Oligonucleotide primers used for cDNA cloning, qRT-PCR, preparation of reporter constructs, and ChIP. fwd, forward primer; rev, reverse primer.
