# Supplementary figures and images for: EVI1 promotes tumor growth via transcriptional repression of MS4A3
Source: J Hematol Oncol. 2015 Mar 21;8:28. doi: 10.1186/s13045-015-0124-6 (PMC4389965; doi:10.1186/s13045-015-0124-6)

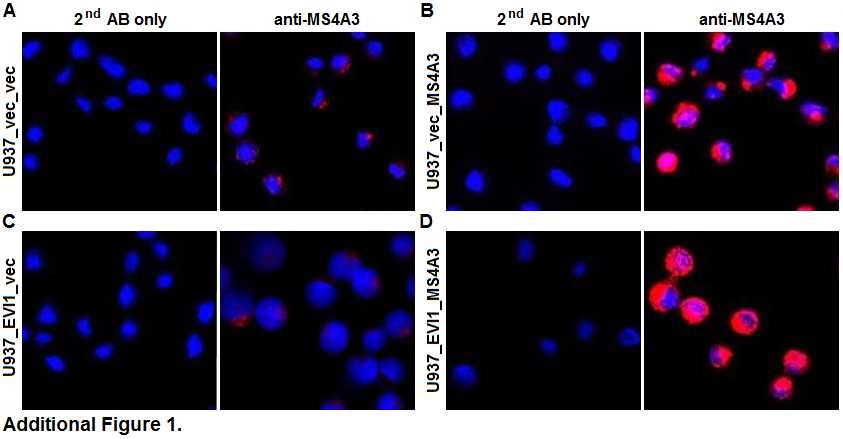

Supplement: Additional file 2: Figure S1. — Confirmation of ectopic expression of MS4A3 in transduced U937_EVI1 and U937_vec cells. Immunofluorescence analysis of MS4A3 expression in U937_vec_vec (A), U937_vec_MS4A3 (B), U937_EVI1_vec (C), and U937_EVI1_MS4A3 (D) cells. Endogenous MS4A3 was observed in U937_vec_vec and U937_EVI1_vec cells, and strong ectopic MS4A3 expression was present in U937_vec_MS4A3 and U937_EVI1_MS4A3 cells. [file 13045_2015_124_MOESM2_ESM.zip › 13045_2015_124_MOESM2_ESM.bmp]

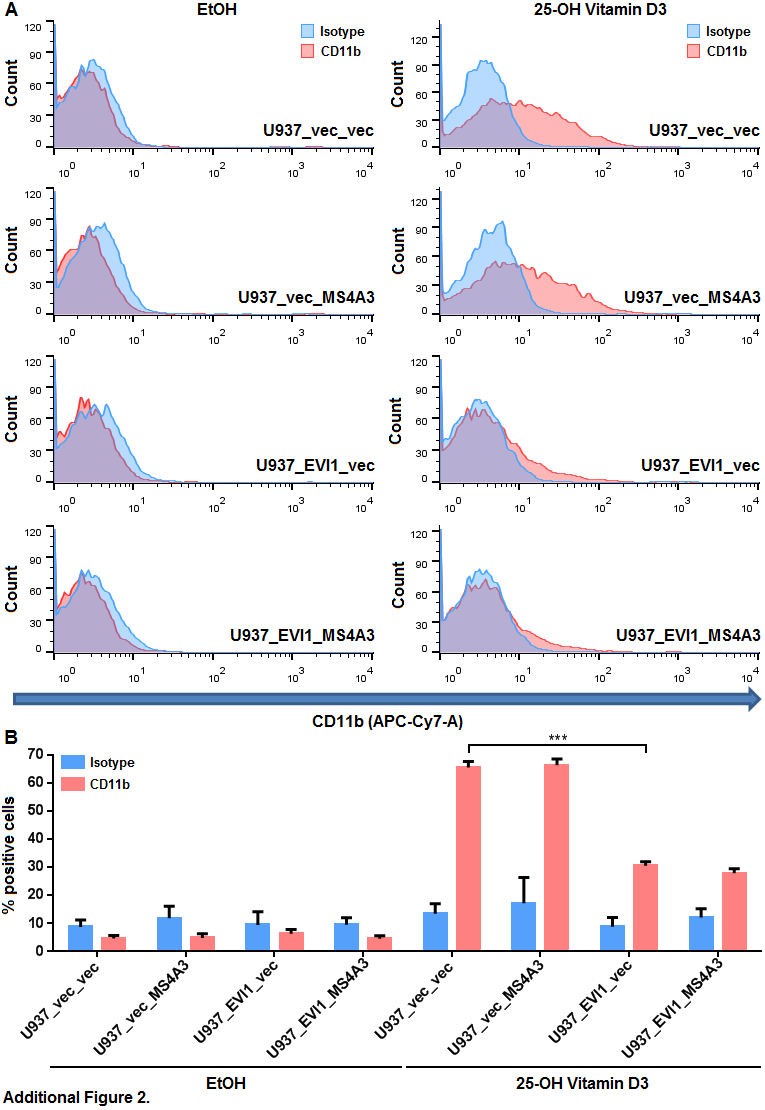

Supplement: Additional file 3: Figure S2. — EVI1 inhibits 25-OH Vitamin D3 induced myelomonocytic differentiation of U937 cells, but ectopic expression of MS4A3 has no impact on this process. U937_vec_vec, U937_vec_MS4A3, U937_EVI1_vec, and U937_EVI1_MS4A3 cells were treated with EtOH (solvent) or 25-OH Vitamin D3 for 5 days, and the extent of myelomonocytic differentiation was determined by flow cytometry after staining for CD11b. (A) Histograms from a representative experiment. Blue areas, isotype control antibody; red areas, APC-Cy7 conjugated CD11b antibody. (B) Summary of flow cytometric data from three independent biological replicate experiments. Mean percentages of positive cells + SEMs are shown. ***p <0.001; (Student’s t-test, two-tailed). [file 13045_2015_124_MOESM3_ESM.zip › 13045_2015_124_MOESM3_ESM.bmp]

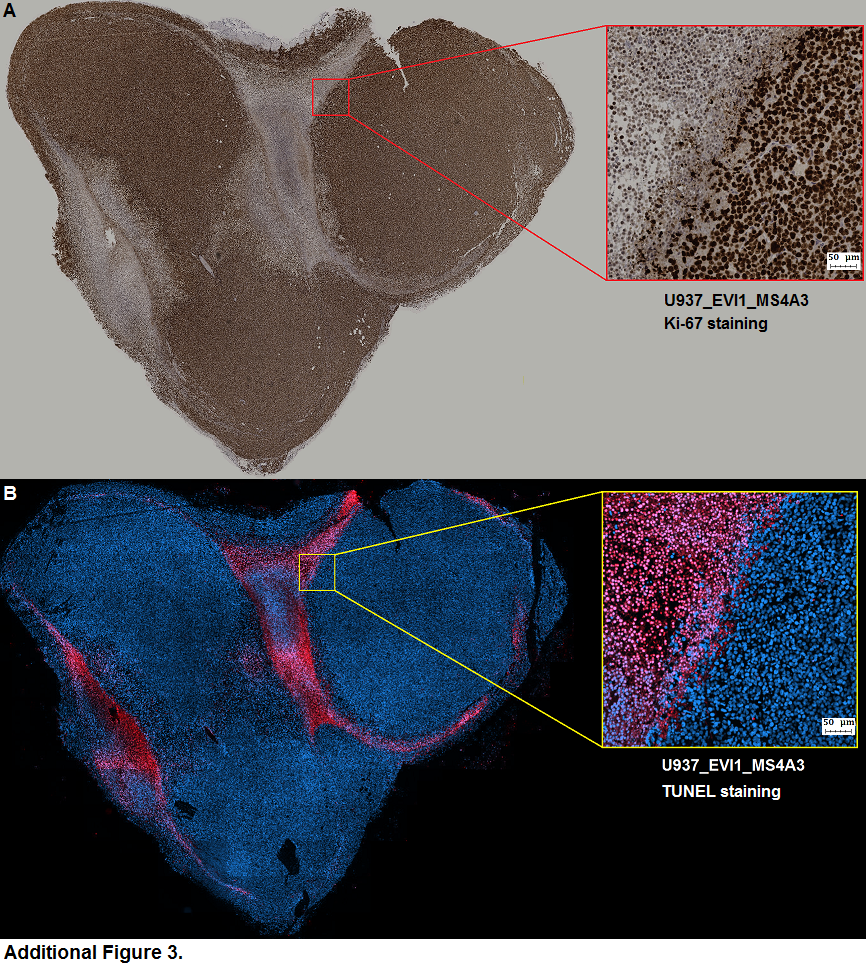

Supplement: Additional file 4: Figure S3. — Nuclear localization of Ki-67 and of double strand break containing DNA identified through TUNEL staining. Magnifications from one of the tumors shown in Figure 4B. (A) Immunohistochemical staining for Ki-67; (B) TUNEL staining. Scale bar, 50 μm. [file 13045_2015_124_MOESM4_ESM.zip › 13045_2015_124_MOESM4_ESM.bmp]
